# A Case of VEXAS: Vacuoles, E1 Enzyme, X-linked, Autoinflammatory, Somatic Syndrome With Co-existing DNA (Cytosine-5)-Methyltransferase 3A Mutation Complicated by Localized Skin Reaction to Tocilizumab and Azacitidine

**DOI:** 10.7759/cureus.39906

**Published:** 2023-06-03

**Authors:** Jordan Estes, Matthew Malus, Lorena Wilson, Peter C Grayson, Mehrdad Maz

**Affiliations:** 1 Department of Medicine, University of Kansas Medical Center, Kansas City, USA; 2 Department of Medicine, Division of Allergy, Clinical Immunology, and Rheumatology, University of Kansas Medical Center, Kansas City, USA; 3 National Human Genome Research Institute, National Institutes of Health, Bethesda, USA; 4 National Institute of Arthritis and Musculoskeletal and Skin Diseases, National Institutes of Health, Bethesda, USA

**Keywords:** myelodysplastic syndrome, azacitidine, tocilizumab, dnmt3a, uba1, vexas

## Abstract

Vacuoles, E1 enzyme, X-linked, autoinflammatory, somatic (VEXAS) syndrome is a recently identified autoinflammatory condition with a correlating missense somatic mutation of the X chromosome. Here we present a unique case of a patient with VEXAS syndrome with coinciding ubiquitin-like modifier activating enzyme 1 (UBA1) and DNA (cytosine-5)-methyltransferase 3A (DNMT3A) mutations who developed cutaneous and systemic reactions to tocilizumab and azacitidine therapy, respectively.

## Introduction

VEXAS (Vacuoles, E1 enzyme, X-linked, Autoinflammatory, Somatic) syndrome is a recently identified severe adult-onset inflammatory disorder due to an acquired missense somatic mutation in the ubiquitin-like modifier activating enzyme 1 (UBA1) gene of the X chromosome. Described in late 2020, VEXAS syndrome was first characterized when gene analysis revealed a UBA1 mutation among 25 males with similar phenotypes [1]. These patients had overlapping inflammatory and hematologic manifestations, including fever, chondritis, vasculitis, cytopenia, neutrophilic dermatosis, and thromboembolism. Bone marrow biopsies demonstrated cytoplasmic vacuoles in the erythroid and myeloid precursor cells, the age of onset was in the fifth decade of life or later, and there was high disease-related mortality [1,2].

As of March 2022, more than 100 cases of VEXAS syndrome have been reported. The diagnosis often coincides with a previously diagnosed rheumatologic disease, most commonly relapsing polychondritis, and hematologic aberrations, most commonly myelodysplastic syndrome (MDS) [1]. These patients often have profound hematologic abnormalities that can progress to hematologic malignancy. Myelodysplastic syndrome is often present with transfusion-dependent cytopenias such as anemia. Overall treatment and control of inflammatory symptoms is challenging, with resistance to immunomodulating and immunosuppressive therapies known to be a common feature [3]. Despite the expanding number of case reports with VEXAS syndrome, a coinciding DNA (cytosine-5)-methyltransferase 3A (DNMT3A) mutation has been rarely reported [3-5]. Local skin reactions to therapeutic injection sites have also been infrequently documented outside of a known association with anakinra injection site reactions [1].

Here, we present a unique case of a patient with VEXAS syndrome with coinciding UBA1 and DNA (cytosine-5)-methyltransferase 3A (DNMT3A) mutations who developed cutaneous and systemic reactions to tocilizumab and azacitidine therapy, respectively.

## Case presentation

The patient is a 62-year-old Caucasian male with a history of recurrent deep vein thromboses and pulmonary embolisms on anticoagulation, Graves’ disease, hypertension, hyperlipidemia, coronary artery disease, systolic heart failure, and atrial fibrillation who was initially referred to our rheumatology clinic by an outside rheumatologist. Social history was pertinent for smoking tobacco for over 40 years. The patient began having bilateral ankle and shin pain in early 2017 and was diagnosed with relapsing polychondritis by his physician after he developed recurrent auricular inflammation. He was initially treated with methotrexate with little relief, mycophenolate mofetil without relief, and subsequently adalimumab but continued to require 30 to 40 mg of prednisone daily. The patient had developed deep vein thrombosis and pulmonary embolism in 1999 as well as a recurrent deep vein thrombosis and pulmonary embolism in 2017 despite anticoagulation with apixaban. He developed transfusion-dependent anemia in 2019, and a bone marrow biopsy confirmed a diagnosis of MDS for which he was started on the hypomethylating agent azacitidine.

At the initial rheumatology consult visit in 2020, he was noted to have continued intermittent auricular chondritis, oligoarticular inflammatory arthritis, periorbital edema, and fatigue when prednisone was tapered below a dose of 30 mg daily. A prior skin biopsy of the lower extremity was consistent with leukocytoclastic vasculitis with positive perivascular staining for IgA, IgM, and C3. A bone marrow biopsy in December 2020 was consistent with evolving MDS, with evidence of fibrosis and blasts less than 5%. Next-generation sequencing was positive for DNMT3A mutation.

Laboratory studies at the initial rheumatology visit demonstrated normocytic anemia and thrombocytopenia. Dilute Russell's viper venom time was elevated, but the repeat was normal. Antinuclear antibodies titer was elevated. Erythrocyte sedimentation rate and C-reactive protein were both elevated. Negative autoimmune studies included antibodies to double-stranded DNA, SSA, SSB, Smith, RNP, Jo 1, beta-2 glycoprotein IgG and IgM, phospholipid IgG and IgM, mitochondrial, smooth muscle, rheumatoid factor, cyclic citrullinated peptide, myeloperoxidase, proteinase 3, complement 3, and complement 4. Evaluation of antineutrophil cytoplasmic antibody demonstrated an elevated atypical p-anti-neutrophil cytoplasmic antibody (p-ANCA) titer. Serum protein electrophoresis showed paraproteinemia with an M spike in the gamma region and elevated kappa, lambda, and kappa/lambda ratio. A full list of laboratory values is shown in Table 1.

**Table 1:** Initial laboratory evaluation DRVVT = dilute Russel's viper venom time, anti-SSA/SSB = anti-Sjogren’s syndrome-related antigen A and B, anti-RNP = anti-ribonucleoprotein, anti-CCP = anti-cyclic citrullinated peptide

Laboratory Evaluation		
	Value	Reference range
Hemoglobin	8.8	13.5 - 16.5 (g/dL)
Leukocytes	1.1	4.5 - 11.0 (k/uL)
Platelets	81	150 - 400 (k/uL)
Creatinine	0.84	0.4 - 1.24 (mg/dL)
DRVVT*	1.34	<1.20
Repeat DRVVT	1.17	<1.20
Antinuclear antibodies	1:160 (speckled)	<80
Erythrocyte sedimentation rate	22	<20 mm/h
C-reactive protein	30.7	<8.0 mg/L
Anti-double-stranded DNA	<10	<10
Anti-SSA*	<0.2	<1.0
Anti-SSB*	<0.2	<1.0
Anti-Smith	<0.2	<1.0
Anti-RNP*	<0.2	<1.0
Anti-Jo 1	<0.2	<1.0
Anti-beta-2 glycoprotein IgG	<9.4	<15.0
Anti-beta-2 glycoprotein IgM	<9.4	<15.0
Anti-Phospholipid IgG	<9.4	<15.0
Anti-Phospholipid IgM	<9.4	<15.0
Anti-mitochondrial	Negative	Negative
Anti-smooth muscle	Negative	Negative
Rheumatoid factor	<10	<25
Anti-CCP*	<0.5	<3.0
Anti-myeloperoxidase	<0.2	<1.0
Anti-proteinase 3	<0.2	<1.0
Complement 3	114	88 – 200 (mg/dL)
Complement 4	24	10 – 49 (mg/dL)
Antineutrophil cytoplasmic antibody	Atypical p-ANCA	Negative
Atypical p-antineutrophil cytoplasmic antibody	>1:640	<1:20
Serum protein electrophoresis	Paraproteinemia with M spike in gamma region	Negative

Given his history and manifestations of polychondritis and myelodysplasia, the leading differential became VEXAS syndrome, a recently recognized entity at the time. Serum samples to evaluate for VEXAS were sent to the National Institutes of Health (NIH), which demonstrated the characteristic UBA1 mutation. Sanger sequencing of the UBA1 gene in peripheral blood revealed a mutation at p.Met41 (p.Met41Thr, c.122T>C). The patient was enrolled in the registry and for a tentative VEXAS treatment protocol at the NIH.

**Figure 1:**
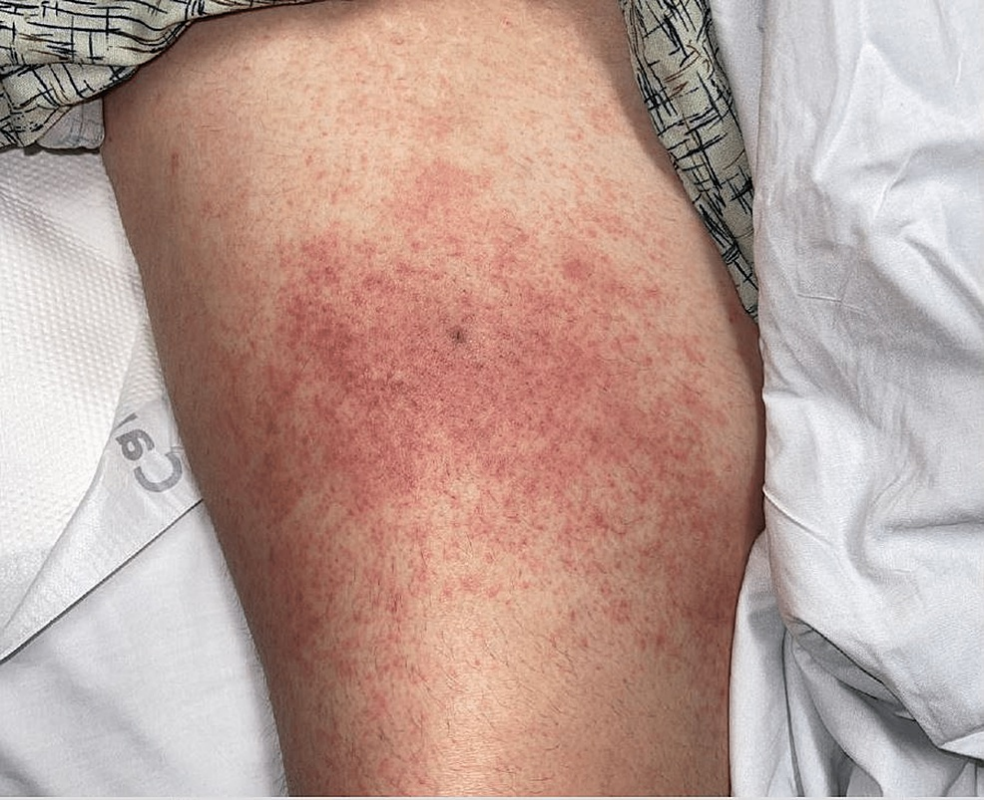
Subcutaneous reaction to azacitidine injection (right anterior thigh)

**Figure 2:**
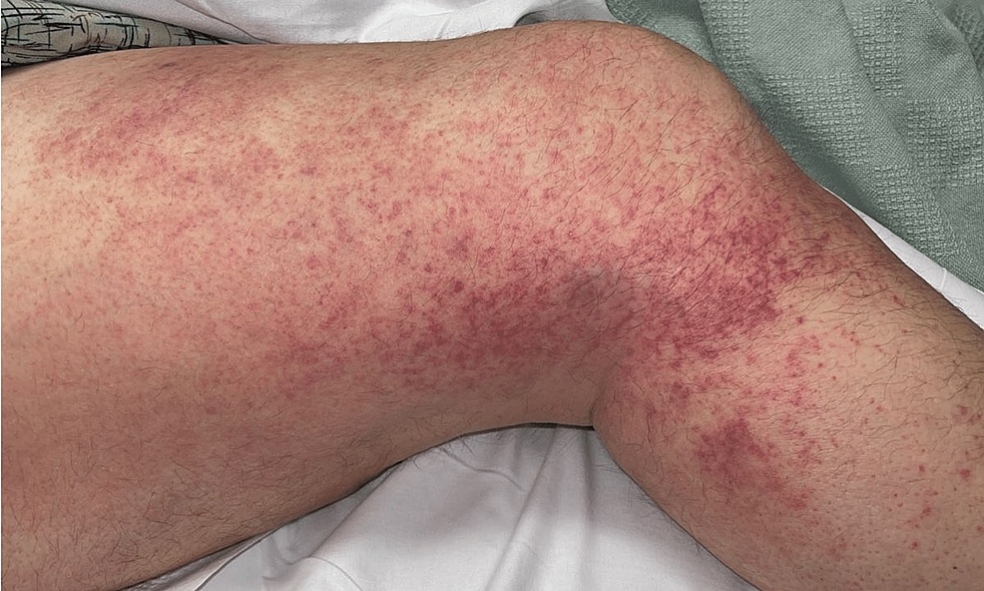
Subcutaneous reaction to azacitidine injection (right lateral leg)

The patient began therapy with tocilizumab in June 2021. He developed a painful dermatitis of his right thigh over the injection site of tocilizumab, for which he was hospitalized. This occurred the day after the fifth dose, which was administered over a period of several months due to interruptions in therapy secondary to repeat infections of different organ systems. Skin biopsy at that time demonstrated histiocytoid neutrophilic dermatosis, known to be a VEXAS syndrome manifestation. However, initially, it was thought he may have developed a cutaneous allergic reaction to tocilizumab, and it was thus discontinued. One month later, he again developed dermatitis around the injection site of azacitidine (Figures 1, 2), which had been restarted after a several-month hiatus. One month later, azacitidine was administered intravenously by his local medical team at an outside hospital, after which the patient developed an anaphylactic shock, so the azacitidine was also discontinued.

As of this writing, the patient remains glucocorticoid-dependent, requiring moderate doses of prednisone. Recurrent inflammatory flares coincided when prednisone was tapered below 25 mg daily. He was subsequently referred to a bone marrow transplant team within our institution to be evaluated for a hematopoietic stem cell transplant. Repeat bone marrow biopsy demonstrated MDS with ringed sideroblasts, hypercellular marrow, multilineage dysplasia, and 1% blasts. There was vacuolation in erythroid and granulocyte precursor cells.

## Discussion

VEXAS syndrome is an adult-onset systemic inflammatory syndrome, which is strongly linked to progressive bone marrow failure and carries a high rate of mortality and morbidity. It is caused by a missense mutation in the UBA1 gene on the X chromosome, and thus primarily affects males [1]. The UBA1 gene is responsible for encoding the E1-activating enzyme, which is required for all cellular ubiquitin signaling and is a key regulator in protein homeostasis [6]. Most VEXAS mutations occur at p.41M, which is where the cytoplasmic form of E1-ubiquitin ligase is encoded [2]. Ubiquitination is key in the regulation of the body’s immune response, and its disruption leads to an impaired ubiquitin-proteasome system, causing an accumulation of unfolded proteins and subsequent inflammasome activation with an uncontrolled inflammatory response [2,6]. This cascade includes the upregulation of several cytokines such as interleukin-1 (IL-1), interleukin-6 (IL-6), and tumor necrosis factor (TNF) [4].

Common features include fever, chondritis, neutrophilic dermatosis, and vasculitis. As these features are inflammatory in nature, VEXAS syndrome is often first diagnosed as one of several rheumatological processes, including relapsing polychondritis, Sweet syndrome, giant-cell arteritis, and polyarteritis nodosa [1,2]. Our patient demonstrated each of these features over a several-year timespan and was initially diagnosed with relapsing polychondritis in the setting of recurrent auricular chondritis.

Hematologic abnormalities are part of the VEXAS syndrome and range from macrocytic anemia to overt hematologic malignancy. To date, virtually all patients diagnosed with VEXAS syndrome have vacuoles in myeloid and precursor cells, although this is not pathognomonic, as these vacuoles can be seen in other states such as malignancy, copper deficiency, and alcoholism [2,4]. Our patient indeed did have these characteristic vacuoles in erythroid and granulocyte precursor cells. MDS is also very common, occurring in about 30% of VEXAS patients [4]. The link between MDS and systemic inflammatory disorders, in general, is well known. The underlying mechanism for this association is hypothesized to include abnormalities in myeloid cells, which are mediators in the innate immune system, and dysregulation of cytokines, which assist in normal inflammatory immune response as well as the differentiation of hematopoietic stem cells [7].

While MDS is relatively common in VEXAS patients, mutations outside of the characteristic UBA1 have less commonly been reported. In our case, the patient had a known DNMT3A mutation found on next-generation sequencing. DNMT3A is an important enzyme involved in de novo DNA methylation, and it is hypothesized the DNMT3A gene plays a special role in preventing malignancies [8]. It is well-established that DNMT3A gene mutations carry an increased risk for an assortment of hematological malignancies [4,8]. Our patient had severe, transfusion-dependent MDS, and the severity of his MDS may, in part, be attributed to his co-existing DNMT3A mutation. Per our review, a coinciding DNMT3A mutation with VEXAS syndrome has been reported in the literature in 10 other patients, making it a relatively uncommon association [4,5,9-11].

Skin involvement is the second most common clinical symptom of VEXAS syndrome [1]. The hallmark histopathology is neutrophilic dermatosis with myeloid cell infiltration, often with concurrent leukocytoclastic vasculitis. This can manifest as tender red or violaceous papules as well as erythematous infiltrated plaques and nodules [12]. Less commonly reported are localized skin reactions, as was the case in our patient, who developed erythematous, painful plaques after separate administration of tocilizumab and azacitidine. The original study by Beck et al reported that eight out of 13 patients developed a severe localized cutaneous reaction when treated with the IL-1 antagonist anakinra [1]. There are a few other scattered reports of similar reactions in VEXAS patients to anakinra [10,13-15]. Cutaneous injection-site reactions are known as possible adverse events with biologics in patients without VEXAS syndrome [16,17]. To our knowledge, there have not been other reports of VEXAS patients with injection site reactions to either tocilizumab or azacitidine. The fact our patient had reactions to both tocilizumab and azacitidine is unique and perhaps may be explained by an overall heightened inflammatory state, although the underlying pathophysiology is unclear. A pathergy-like phenomenon could be a potential explanation, although the patient subsequently had a systemic reaction to a trial of intravenous azacitidine. Possible solutions for VEXAS patients experiencing injection site reactions could include desensitization therapy or intravenous administration. Intravenous administration should be performed under close observation due to the risk of an anaphylactic response, as was the case with our patient.

Treating VEXAS syndrome has proven to be difficult. In fact, lack of response to immunomodulating and immunosuppressive agents appears to be a hallmark trait of the disease [1,4]. Despite the knowledge that UBA1 mutation leads to the upregulation of inflammatory cytokines such as TNF, IL-1, and IL-6, antagonistic therapies have not yet yielded favorable responses [1,4]. Oral glucocorticoids remain the mainstay of treatment, with moderate to high daily doses and a propensity for inflammatory flares when attempting to taper down the treatment. There have been more promising, albeit scattered, reports on steroid-sparing therapies including IL-6, TNF inhibitors, as well as methotrexate, mycophenolate mofetil, azathioprine, cyclosporine, and tacrolimus [4]. One retrospective series showed azacitidine and Janus kinase inhibitors to be the most promising therapies, although the sample size was small [18]. Even after starting anti-IL-6 therapy with tocilizumab, our patient had persistent cytopenias and recurrent inflammatory flares when prednisone was tapered below a daily dose of 25 mg. Many patients will need evaluation for allogeneic stem cell transplantation (ASCT) as a possible definitive therapy [1]. Although data are limited regarding ASCT efficacy, a recent study was promising, with five out of six patients in complete remission at three, five, 32, 37, and 38 months after ASCT, respectively. The sixth patient died from infectious complications [19]. Although bone marrow transplant is potentially curative, there is an inherent risk for complications and mortality, which, in turn, highlights the need to define the highest-risk VEXAS patients that may benefit from ASCT the most.

VEXAS syndrome currently carries a poor prognosis overall, with disease-related mortality estimates ranging from 18% to 50% (Abstract: Ferrada M, Savic S, Alessi H, et al. Genotype and Transfusion Dependence Predicts Mortality in VEXAS Syndrome, a Newly Described Disease With Overlap Inflammatory and Hematologic Features. ACR Convergence; November 8, 2021, [1,3]). Certain subsets of patients may have an increased risk of mortality. A recent study by Ferrada et al. included 73 patients with VEXAS syndrome and demonstrated a relationship between genotype, transfusion-dependent cytopenia, and survival. Specifically, a valine mutation at the p.Met41 location was associated with higher mortality (50%) compared to leucine (18%) or threonine (22%) mutations. Transfusion dependence was the only other independent risk factor associated with mortality (Abstract: Ferrada M, November 8, 2021). Classification of a higher-risk subset could help identify patients who would benefit from timely referral for bone marrow transplant evaluation. Our patient had a threonine mutation, which is associated with lower mortality (22%), but he was transfusion dependent and therefore does have a high-risk feature. He is appropriately being evaluated for a bone marrow transplant.

## Conclusions

In conclusion, this case adds to the growing body of evidence correlating a complex picture of inflammatory and hematologic conditions to the causative somatic mutation of UBA1. It also adds to the evidence of a disease that is refractory to steroid-sparing therapy; in this case, tocilizumab. Given the high disease-related mortality, VEXAS syndrome needs to be considered in middle-aged to elderly patients who present with a constellation of multisystem inflammatory and hematologic disease states that are resistant to target-specific anti-rheumatic therapies. This case highlights the importance of a multi-disciplinary approach for the ongoing care of VEXAS syndrome patients, with special consideration for bone marrow transplant referrals in more high-risk patients.
